# Clinic Value of Blood Urea to Creatinine Ratio in Heart Failure Patients: A Systematic Review

**DOI:** 10.1002/clc.70344

**Published:** 2026-05-25

**Authors:** Miguel Cabanillas‐Lazo, Roger A. Sernaqué‐Mechato, Alvaro Montes‐Baldarrago, Jeancarlo Velazco Muñoz, Valeria Loja Zapata, Ivan Alegre‐Cordero, Carlos Quispe‐Vicuña, Frank Mayta‐Tovalino

**Affiliations:** ^1^ Grupo de Investigación Neurociencias, Metabolismo, Efectividad Clínica y Sanitaria (NEMECS) Universidad Científica del Sur Lima Perú; ^2^ Hospital Santa Rosa Lima Perú; ^3^ Facultad de Medicina Humana, Universidad Continental Lima Perú; ^4^ Sociedad Científica de San Fernando Lima Perú; ^5^ Facultad de Medicina de San Fernando Universidad Nacional Mayor de San Marcos Lima Perú; ^6^ Vicerrectorado de Investigacion Universidad San Ignacio de Loyola Lima Perú

**Keywords:** blood urea nitrogen, creatinine, heart failure, systematic review

## Abstract

**Aims:**

To synthesize the existing evidence on the association of BCR with clinical outcomes in patients with heart failure.

**Methods:**

A comprehensive and systematic search of observational studies was conducted across five major databases—PubMed, Embase, Scopus, Web of Science, and Google Scholar—covering all records up to February 2024. The primary outcomes of interest included mortality, rehospitalization, cardiovascular events, and renal complications. To evaluate the methodological quality, the Newcastle–Ottawa scale was applied, allowing for the structured assessment of bias across the selection, comparability, and outcome domains. Additionally, the certainty of the evidence was appraised using the GRADE framework.

**Results:**

Twenty studies including 21 397 HF patients were analyzed. For ≥ 1‐year mortality (11 661 participants, 11 studies), hazard ratios ranged from 1.30 to 2.19, with the strongest association in HF with preserved ejection fraction (HR: 3.28, 95% CI: 2.00–5.38) with very low certainty. It also correlated with worse NYHA class IV versus II (MD: 12.03, 95% CI: 10.54–13.52) and increased risk of 1‐month major adverse cardiovascular events (OR: 2.47, 95% CI: 1.01–6.01), both with very low certainty. Most included cohort studies were rated as low risk of bias, with only two studies at moderate risk and one study at high risk.

**Conclusion:**

Elevated BCR is associated with adverse outcomes in patients with heart failure. However, its discriminative performance appears limited, and its incremental value over established biomarkers such as NT‐proBNP remains unclear. Further prospective studies are required to determine its clinical utility.

## Graphical abstract

Elevated blood urea nitrogen‐to‐creatinine ratio (BCR) is associated with increased mortality, rehospitalization, and adverse clinical outcomes in heart failure patients; however, its prognostic accuracy is limited and its added value over established biomarkers remains uncertain.

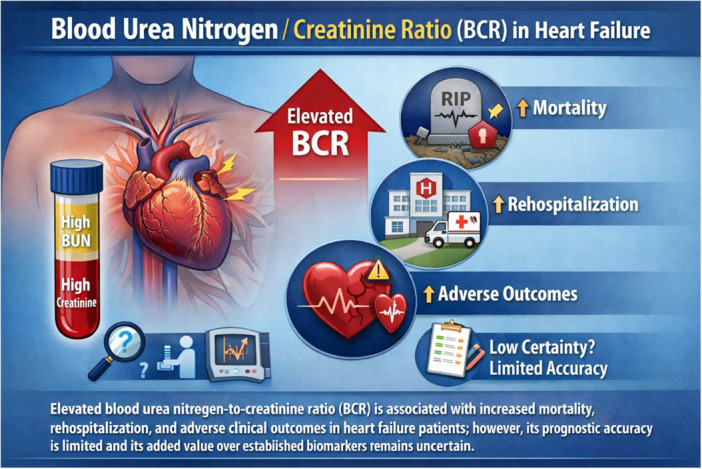

## 1 Introduction

Heart failure (HF) is a clinical syndrome characterized by symptoms or signs of cardiac dysfunction, such as dyspnea and fatigue, which are evidenced by elevated natriuretic peptide levels or cardiopulmonary and systemic congestion [1]. HF is increasingly prevalent, affecting over 64 million people worldwide, with costs projected to reach 53.1 billion dollars by 2030 [2].

Heart Failure (HF) patients experience a mortality rate that is more than three times that of the general population [3, 4]. Patients with HF have a presentation of comorbidities that differs by gender; women present with atrial fibrillation, valvular heart disease, and sleep apnea, and men present with hypertension, diabetes, and depression [2]. The presence of comorbidities, chronic disease state, and frequent hospitalizations contribute to decreased health‐related quality of life. HF patients also experience the risk of being unemployed, facing early retirement, and long‐term absences from work [5].

For this reason, biomarkers include N‐terminal prohormone brain natriuretic peptide (NT‐proBNP), biglycan, YKL40 protein, cardiac troponin, and genes such as Heat2 and COL1A1 were suggested to correlate with HF severity and mortality [6, 7]. However, developing and validating this prediction models can be costly due to data collection and analysis requirements, especially when involving tissue acquisition or allelic variant screening instead of blood testing [8].

Blood urea nitrogen (BUN) and creatinine (Cr) have been of interest in assessing the severity of HF due to their role as indicators of renal hypoperfusion and its relation to low cardiac output or congestion, although both present individual limitations due to external and metabolic influences [9, 10]. In this sense, the BUN‐to‐Cr ratio (BCR) reduces confounding factors compared to individual BUN or Cr and has been used to evaluate the prognosis of poor outcomes in conditions such as HF. Upon admission, an increase in BCR has been significantly linked to in‐hospital death, long‐term mortality, and comorbidity in patients with acute ischemic stroke, representing a marker of early neurological deterioration via dehydration [10]. Similarly, higher BCR is associated with in‐hospital mortality in acute respiratory distress syndrome, a manifestation of organ failure after trauma [11].

Therefore, BCR has been proposed as a potential biomarker, although its prognostic performance and added value over established markers remain uncertain. The aim of this systematic review is to summarize the current knowledge regarding the capacity of BCR to predict outcomes in HF patients.

## 2 Methods

This systematic review was conducted in accordance with the guidelines outlined in the Preferred Reporting Items for Systematic Reviews and Meta‐Analyses (PRISMA) framework [12]. The study protocol was prospectively registered in the PROSPERO database (registration ID: CRD42024518113), ensuring methodological transparency, reproducibility, and adherence to internationally recognized standards for evidence synthesis.

### 2.1 PECO Question

This review sought to address the following clinical question: *What is the prognostic relevance of the blood urea to creatinine ratio in patients diagnosed with heart failure?* The aim was to explore whether this biochemical marker offers meaningful insight into patient outcomes and disease severity within this population.

### 2.2 Eligibility Criteria

Studies were considered eligible for inclusion if they met the following conditions: (1) involved adult participants (aged over 18 years) diagnosed with any form of heart failure; (2) reported blood urea to creatinine ratio (BCR) values measured at the time of hospital admission; and (3) employed an analytical observational design. Articles were excluded if they were narrative or systematic reviews, conducted in non‐human subjects, or presented as case reports, conference abstracts, or letters to the editor.

### 2.3 Search of Studies

A thorough literature search was performed across five major databases—PubMed, Embase, Scopus, Google Scholar, and Web of Science—encompassing all publications available up to February 2024. The initial search algorithm developed for PubMed was thoughtfully adapted to align with the indexing systems and search functionalities of each platform (see Supporting Information S1). No filters were applied regarding language or publication date, allowing for a broad and inclusive identification of relevant studies. To further strengthen the comprehensiveness of the review, the reference lists of all selected articles were manually examined, and additional studies meeting the eligibility criteria were incorporated.

### 2.4 Outcomes

The outcomes were focused on clinical severity and prognosis. The primary outcomes were mortality and rehospitalization. Secondary outcomes were categorized into cardiac‐related (MACE, NYHA, ejection fraction, and congestion) and renal‐related outcomes (AKI, renal improvement, hyponatremia, eGFR, and urinary volume).

### 2.5 Study Selection

Search results retrieved from electronic databases were initially managed using EndNote X9, where duplicate entries were identified and subsequently transferred to Rayyan (https://rayyan.qcri.org/) for screening. Two independent reviewers (A.M.B. and V.L.Z.) conducted the selection process, evaluating full‐text articles against the predefined inclusion criteria. Any disagreements were resolved through discussion and, when necessary, with input from a third reviewer (M.C.L.) acting as the arbitrator. The full list of excluded studies, along with the reasons for exclusion, is available in Supporting Information S2.

### 2.6 Data Extraction

The process of data extraction was carried out independently by two reviewers (A.M.B. and J.V.M.) using a standardized extraction form. Any discrepancies between the reviewers were resolved through consultation with a third author (M.C.L.), who served as the adjudicator. The following variables were systematically retrieved from each study: title, first author, year of publication, study design, country of origin, sample size, sex distribution, age of participants, timing of sample collection, mean or median BCR values, crude and adjusted measures of association, sensitivity and specificity estimates, and the type and operational definition of reported outcomes. When additional information was required, the corresponding authors were contacted via email to obtain clarification or Supporting data.

### 2.7 Risk of Bias Assessment

The evaluation of the study quality was performed using the Newcastle–Ottawa scale (NOS) [13] by two independent reviewers (A.M.B. and J.V.M.). Any discrepancies in the scoring were resolved through discussion with a third reviewer (M.C.L.). This instrument is specifically designed to appraise the methodological rigor of nonrandomized studies, focusing on three core domains: selection of participants, comparability between groups, and assessment of outcomes or exposures. Each domain included specific subcriteria, and the studies were rated using a star‐based scoring system. A total score of 6 or higher was interpreted as low risk of bias (high methodological quality), scores between 4 and 5 indicated moderate risk, and scores below 4 were classified as high risk of bias.

### 2.8 Synthesis of Evidence

Although a meta‐analysis was initially planned for each outcome, the synthesis strategy was subsequently adapted to a narrative approach. This decision was driven by two key limitations encountered during the review process: the limited availability of extractable data across studies and the presence of substantial clinical heterogeneity, particularly in relation to study populations, exposure definitions, and outcome measures. These constraints precluded meaningful statistical pooling and warranted a descriptive integration of the findings to preserve the analytical rigor. A narrative synthesis combines evidence from multiple studies using different formats including textual descriptions, tabulated data, and visual representations to create a thorough overview of the available evidence. To achieve this, we employed the framework proposed by Popay et al. [14]: (1) explaining the underlying physiopathological mechanisms that could explain the observed results; (2) developing a textual summary of the characteristics of the included studies; (3) ordering and summarizing the results through a table; and (4) assessing the certainty of the evidence using the GRADE system.

To enhance a more cohesive synthesis of the findings, the continuous variables that were reported as medians with interquartile ranges (IQRs) were transformed into means and standard deviations (SDs) using the approach recommended by Wan et al. The primary measures of association retrieved from the included studies were hazard ratios (HR), risk ratios (RR), and odds ratios (OR), and studies provided a 95% confidence interval (CI) for each of these measures.

### 2.9 Certainty of the Evidence

The certainty of the narratively synthesized findings was independently evaluated by two reviewers (M.C.L. and C.Q.V.) using the Grading of Recommendations, Assessment, Development, and Evaluation (GRADE) [15, 16] approach tailored for narrative synthesis. This framework considers five key domains: methodological limitations of the included studies (risk of bias), imprecision (related to sample size and confidence intervals), indirectness (applicability and generalizability of the evidence), inconsistency (variability across study results), and potential publication bias, as outlined in the GRADE Handbook. Publication bias was assessed qualitatively due to the absence of meta‐analysis, considering the number of available studies, study size, selective reporting, and the likelihood of unpublished negative results, in accordance with GRADE guidance. Based on these criteria, the overall certainty of the evidence was classified as high, moderate, low, or very low.

## 3 Results

### 3.1 Selection of Studies

**FIGURE 1:**
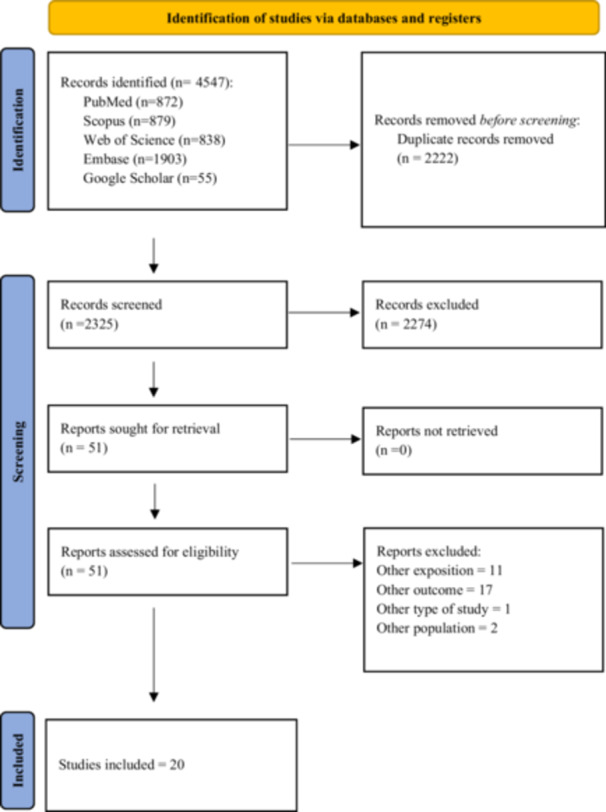
PRISMA flowchart of included studies.

The systematic search yielded a total of 4547 records. Following the removal of 2222 duplicates, 2325 unique titles and abstracts were screened for relevance. Of these, 2274 were excluded based on the predefined eligibility criteria. Ultimately, 20 studies met the inclusion criteria and were incorporated into the final review (Figure 1) [17, 18, 19, 20, 21, 22, 23, 24, 25, 26, 27, 28, 29, 30, 31, 32, 33, 34, 35, 36].

### 3.2 Study Characteristics

The included studies involved 20 cohort studies. Altogether, the sample comprised 21 397 patients with HF. The average age ranged from 56 to 82 years and came from studies conducted across Asia, Europe, and North America. Most studies focused on prognostic outcomes with follow‐up durations varying from 1 month to more than 6 years. Supporting Information S3 summarizes the main characteristics of the included studies.

### 3.3 Risk of Bias Assessment

The methodological quality of the 20 cohort studies included in this review was assessed according to established evaluation criteria. Overall, most studies exhibited a low risk of bias, indicating that their design and reporting met acceptable methodological standards. Nonetheless, two studies, Verbrugge et al. [34] and Kinugawa et al. [24]—presented a moderate risk of bias, mainly related to issues of population representativeness, selection of non‐exposed comparison groups, and the adequacy of follow‐up procedures. In contrast, one study, Schaer et al. [31], was rated as having a high risk of bias due to methodological limitations in defining the study cohorts, ensuring group comparability, and assessing outcomes. Comprehensive details of the quality assessment are provided in Supporting Information S4.

### 3.4 Mortality

Regarding the studies analyzing BCR and mortality, Gotsman et al. [21], in a long‐term follow‐up study of 6.5 years, observed increased mortality across BCR with an adjusted hazard ratio (aHR) of 1.55 (95% CI: 1.11–2.16). Brisco et al. [20], using a cutoff of 13.3, reported that high BCR was associated with increased mortality, with an aHR of 1.30 (95% CI: 1.10–1.40) at 2.8 years. Over a 2.6‐year follow‐up, Parrinello et al. [28] reported a significant association using a cutoff of 25.5, with an aHR of 2.19 (95% CI: 1.21–3.94), while Brisco 1 et al. [19] also found a significant association with mortality, with an aHR of 1.30 (95% CI: 1.10–1.50). Sujino et al. [32] found a significant relationship between higher BCR and 2.1‐year mortality, reporting a crude hazard ratio (cHR) of 1.02 (95% CI: 1.02–1.03).

Kang et al. [23] evaluated patients with heart failure with reduced ejection fraction (HFrEF), mid‐range ejection fraction (HFmrEF), and preserved ejection fraction (HFpEF), using a cutoff of 20.4. They found that higher BCR values were significantly associated with an increased risk of 2‐year mortality in HFpEF (aHR: 3.28; 95% CI: 2.00–5.38), whereas no significant associations appeared in HFrEF (aHR: 1.04; 95% CI: 0.66–1.65) or HFmrEF (aHR: 1.57; 95% CI: 0.99–2.50). Murata et al. [27] demonstrated a significant association using a cutoff of 20.4, with an aHR of 1.80 (95% CI: 1.16–2.80), whereas Wang et al. [35] detected a comparable association with an aHR of 1.89 (95% CI: 1.30–2.74) using a cutoff of 19.4 during a 1.9‐year follow‐up. Massari et al. [25] found a mean difference (MD) of 2.66 (95% CI: 4.81 to 0.51), and using a cutoff of 25, they calculated an aHR of 1.70 (95% CI: 1.00–2.50). They also reported a sensitivity of 56.0%, specificity of 63.0%, and an AUC of 0.61 at 1.5 years.

Josa‐Laorden et al. [22] identified a significant association, reporting an MD of 5.96 (95% CI: 11.64–0.28), and with a cutoff of 50.0, they observed an adjusted odds ratio (aOR) of 2.75 (95% CI: 1.37–5.53). Meanwhile, Rubio‐Gracia et al. [29] found no meaningful MD (0.0; 95% CI: 0.11–0.11); however, using a cutoff of 0.5, they reported a crude hazard ratio (cHR) of 1.41 (95% CI: 0.77–2.60), with a sensitivity of 57.1%, specificity of 53.0%, and an AUC of 0.57 (95% CI: 0.46–0.67). Both studies assessed outcomes at 1 year of follow‐up. Aronson et al. [18] evaluated BCR in quartiles. For quartile 2 (BCR: 16–20), they found an adjusted relative risk (aRR) of 1.10 (95% CI: 0.60–1.80). For quartile 3 (BCR: 21–27), the aRR was 1.00 (95% CI: 0.60–1.60). For quartile 4 (BCR > 27), the aRR increased to 2.30 (95% CI: 1.40–3.80), with a follow‐up of 0.9 years. Finally, Matsue et al. [26] reported an aHR of 1.86 (95% CI: 1.29–2.66) for 6‐month mortality.

### 3.5 Rehospitalization

Kang et al. [23] evaluated the association between BCR and 2‐year rehospitalization. Using a cutoff of 25.5, they reported no significant associations in HFrEF, an aOR of 0.71 (95% CI: 0.43–1.18), or HFmrEF, an aOR of 0.95 (95% CI: 0.58–1.54), while in HFpEF, higher BCR values were associated with an increased risk of rehospitalization, an aOR of 1.78 (95% CI: 1.09–2.93). On the other hand, Matsue et al. [26], with a cutoff between 12.9 and 17.6, reported for 2‐month rehospitalization that patients with lower BCR had an aHR of 0.74 (95% CI: 0.18–3.14), while those with higher BCR showed an aHR of 1.23 (95% CI: 0.81–1.86) (Table 1).

**TABLE 1:** Narrative synthesis of included studies evaluating mortality and rehospitalization of blood urea nitrogen to creatinine ratio (BCR) in heart failure (HF).

Study‐ID	Number of analized patients	BCR mean (SD) in poor outcome	BCR mean (SD) in good outcome	BCR mean difference (CI)	Correlation BCR	BCR cutoff	Effect size	Sensitivity (%)	Specificity (%)	Area under the curve (AUC)	Follow‐up
Mortality											
Gotsman, 2010	355	NR	NR	NR	NR	NR	aHR: 1.55 (95% CI: 1.11–2.16)	NR	NR	NR	6.5 years
Brisco, 2017	4214	NR	NR	NR	NR	13.3	High BCR:aHR: 1.30 (95% CI: 1.10–1.40	NR	NR	NR	2.8 years
Parrinello, 2015	103	NR	NR	NR	NR	25.5	aHR: 2.19 (95% CI: 1.21–3.94)	NR	NR	NR	2.6 years
Brisco, 2013	896	NR	NR	NR	NR	NR	aHR: 1.3 (95% CI: 1.1–1.5)	NR	NR	NR	2.6 years
Sujino, 2019	2090	NR	NR	NR	NR	NR	cHR: 1.02 (95% CI: 1.02–1.03)	NR	NR	NR	2.1 years
Kang, 2023	2099	NR	NR	NR	NR	20.4	HFrEF: aHR: 1.57 (95% CI: 0.99–2.50) HFmrEF: aHR: 1.04 (95% CI: 0.66–1.65) HFpEF: aHR: 3.28 (95% CI: 2.00–5.38)	NR	NR	NR	2 years
Murata, 2018	557	NR	NR	NR	NR	20.4	aHR: 1.80 (95% CI: 1.16–2.80)	NR	NR	NR	1.9 years
Wang, 2023	504	NR	NR	NR	NR	19.4	aHR: 1.89 (95% CI: 1.30–2.74)	NR	NR	NR	1.9 years
Massari, 2020	436	26.33 (9.79)	23.67 (7.44)	−2.66 (95% CI: −4.81 to −0.51)	NR	25	aHR: 1.70 (95% CI: 1.0–2.50)	56.0	63.0	0.61	1.5 years
Josa‐Laorden, 2018	204	54.66 (20.53)	48.70 (15.67)	−5.96 (95% CI: −11.64 –−0.28)	NR	50.0	aOR: 2.75 (95% CI: 1.37–5.53)	NR	NR	NR	1 year
Rubio‐Gracia, 2021	203	0.51 (0.34)	0.51 (0.16)	0.0 (95% CI: −0.11–−0.11)	NR	0.5	cHR: 1.41 (95% CI: 0.77–2.60)	57.1	53.0	0.57 (95% CI: 0.46–0.67)	1 year
Aronson, 2004	541	NR	NR	NR	NR	16.0	Quartile 2 (BRC: 16‐20): aRR: 1.10 (95% CI: 0.60–1.80) Quartile 3 (BCR: 21–27): aRR: 1.00 (95% CI: 0.60–1.60) Quartile 4 (BCR > 27): aRR: 2.30 (95% CI: 1.40–3.80)	NR	NR	NR	0.9 year
Matsue, 2018	1956	NR	NR	NR	NR	NR	aHR: 1.86 (95% CI: 1.29–2.66)	NR	NR	NR	6 months
Rehospitalization											
Kang, 2023	2099	NR	NR	NR	NR	25.5	HFrEF: aOR: 0.71 (95% CI: 0.43–1.18) HFmrEF: aOR: 0.95 (95% CI: 0.58–1.54) HFpEF: aOR: 1.78 (95% CI: 1.09–2.93)	NR	NR	NR	2 years
Matsue, 2018	1956	NR	NR	NR	NR	12.9 –17.6	Low BCR: aHR: 0.74 (95% CI: 0.18–3.14) High BCR: aHR: 1.23 (95% CI: 0.81–1.86)	NR	NR	NR	2 months

Abbreviations: aHR, Adjusted Hazard Ratio; aOR, Adjusted Odds Ratio; aRR, Adjusted Relative Risk; cHR, Crude Hazard Ratio; CI, Confidence interval; HFrEF, Heart Failure with reduced Ejection Fraction; HFmrEF, Heart Failure with mid‐range Ejection Fraction; HFpEF, Heart Failure with preserved Ejection Fraction; NR, Not reported; SD, Standard deviation.

### 3.6 Cardiac Outcomes

Alya et al. [17] evaluated the association between BCR and a 1‐month major adverse cardiovascular event (MACE). Using a cutoff of 16.05, they reported a significant association, with an aOR of 2.47 (95% CI: 1.01–6.01), with a sensitivity of 63.8%, specificity of 56.9%, and an AUC of 62.7 (Table 2).

**TABLE 2:** Narrative synthesis of included studies evaluating the association between the blood urea nitrogen to creatinine ratio (BCR) and cardiac outcomes in heart failure patients.

Study‐ID	Number of analized patients	BCR mean (SD) in poor outcome	BCR mean (SD) in good outcome	BCR mean difference (CI)	Correlation BCR	BCR cutoff	Effect size	Sensitivity (%)	Specificity (%)	Area under the curve (AUC)	Follow‐up
Major Adverse Cardiovascular Event (MACE)											
Alya, 2019	96	NR	NR	NR	NR	16.05	aOR: 2.47 (95% CI: 1.01–6.01)	63.8	56.9	62.7	1 month
Vena cava congestion											
Parrinello, 2015	103	28.2 (9.3)	22.9 (7.0)	−5.3 (95% CI: −8.74 to −1.86)	NR	25.5	aOR: 2.98 (95% CI: 1.23–7.18)	NR	NR	NR	31 months
New York Heart Association (NYHA)											
Xie, 2023	108	NYHA IV 125.11 (2.67)	NYHA II 113.08 (2.27)	12.03 (95% CI: 10.54–13.52)	NR	NR	NR	NR	NR	NR	NR

Abbreviations: aOR, Adjusted Odds Ratio; CI, Confidence interval; NR, Not reported; SD, Standard deviation.

Parrinello et al. [28] found that patients with vena cava congestion had lower BCR compared with those without vena cava congestion with an MD of −5.3 (95% CI: −8.74 to −1.86), with higher values being associated with an aOR of 2.98 (95% CI: 1.23–7.18) over a follow‐up of 31 months (Table 2).

Similarly, Xie et al. [36] reported that patients with stage II of the New York Heart Association (NYHA) presented lower BCR values compared to those with stage IV with an MD of −12.03 (95% CI: −13.52 to −10.54) (Table 2).

### 3.7 Renal Outcomes

For acute kidney injury (AKI), Josa‐Laorden et al. [22] applied a cutoff of 50.0 and reported an AUC of 0.56, while Takaya et al. [33], without adjustment for confounders, found no significant association (cOR: 0.90; 95% CI: 0.57–1.43). Both studies evaluated the outcomes at 1 year of follow‐up. In contrast, Sakr et al. [30] assessed a 3‐day follow‐up and observed no association, with an MD of 1.05 (95% CI: 4.78–2.68) between patients who developed AKI and those who did not and an adjusted odds ratio (aOR) of 1.00 (95% CI: 0.77–1.30).

Verbrugge et al. [34] reported that patients with decompensated hyponatremia had significantly lower BCR compared with those without hyponatremia with an MD of −7.34 (95% CI: −11.71 to −2.97) in 6 months. Kinugawa et al. [24] found higher BCR values in patients with hypernatremic events compared to those without with an MD of 3.0 (95% CI: 0.37–5.63), with hypernatremia being independently associated with adverse events with an aOR of 1.03 (95% CI: 1.02–1.05) at 7 days. Additionally, Schaer et al. [31] identified a negative correlation between natremia and BCR with a Pearson's correlation coefficient of −0.33.

Gotsman et al. [21] found that patients with lower eGFR ( < 35 mL/min/1.73 m²) had higher BCR values compared with those with preserved renal function ( > 53 mL/min/1.73 m²), although the mean difference was not statistically significant, MD of −2.0 (95% CI: −4.22 to 0.22) over 6.5 years of follow‐up. Similarly, Josa‐Laorden et al. [22] observed no significant difference in BCR between patients with reduced renal function (eGFR < 60 mL/min/1.73 m²) and those with preserved function ( > 60 mL/min/1.73 m²), MD of −1.4 (95% CI: −5.84 to 3.04) at 1 year (Table 3).

**TABLE 3:** Narrative synthesis of included studies evaluating the association between the blood urea nitrogen to creatinine ratio (BCR) and renal function in heart failure patients.

Study‐ID	Number of analized patients	BCR mean (SD) in poor outcome	BCR mean (SD) in good outcome	BCR mean difference (CI)	Correlation BCR	BCR cutoff	Effect size	Sensitivity (%)	Specificity (%)	Area under the curve (AUC)	Follow‐up
Acute Kidney Injury (AKI)											
Josa‐Laorden, 2018	204	NR	NR	NR	NR	50.0	NR	NR	NR	0.56	1 year
Takaya, 2015	371	NR	NR	NR	NR	22.1	cOR: 0.9^a^ (95% CI: 0.57–1.43)	NR	NR	NR	1 year
Sakr, 2023	60	21.73 (7.18)	20.68 (7.57)	−1.05 (95% CI: –4.78 to 2.68)	NR	17.4	aOR: 1.00 (95% CI: 0.77–1.30)	NR	NR	0.69	3 days
Glomerular Filtration Rate											
Gotsman, 2010	355	eGFR < 35 23.33 (9.76)	eGFR > 53 21.33 (7.51)	2.0 (95% CI: 0.22–4.22)	NR	NR	NR	NR	NR	NR	6.5 years
Josa‐Laorden, 2018	204	eGFR < 60 50.0 (14.32)	eGFR > 60 48.6 (18.02)	1.4 (95% CI: − 3.04 to 5.84)	NR	NR	NR	NR	NR	NR	1 year

Abbreviations: aOR, Adjusted Odds Ratio; cOR, Crude Odds Ratio; CI, Confidence interval; NR, Not reported; SD, Standard deviation.

^a^ Effect size was not adjusted for confounders.

### 3.8 Certainty Evidence

The overall certainty of the evidence was generally very low, which limits confidence in the synthesized findings. Outcomes related to mortality and rehospitalization at 1 year or beyond showed marked variability in effect estimates and overlapping confidence intervals. As a result, the certainty of evidence for these endpoints was downgraded due to inconsistency and indirectness, largely attributed to variations in follow‐up duration across studies. Evidence concerning cardiac events was also characterized by a high degree of uncertainty, influenced by imprecision and the relatively small sample sizes of the included cohorts. In contrast, renal outcomes demonstrated stronger methodological consistency, achieving a moderate to high level of certainty—particularly in the observed association between elevated blood urea nitrogen (BUN) levels and the risk of rehospitalization. Taken together, the limited homogeneity and statistical precision observed across studies justify the overall low certainty assigned to most evaluated outcomes (Table 4). Publication bias was not detected after qualitative assessment of the evidence. Therefore, no downgrading was applied.

**TABLE 4:** GRADE Summary of Findings (SoF).

Outcomes	№ of participants (studies)	Certainty of the evidence (GRADE)	Anticipated absolute effects	
			Effect size	95% CI
**Mortality or rehospitalization**				
≥ 1 year mortality	11661 (11 observational studies)	⊕◯◯◯ Very low^a^,^b^	Long‐term studies (1.5–6.5 years) reported hazard ratios ranging from 1.30 to 2.19, with 95% CIs between 1.10 and 3.94. In HFpEF patients showed the strongest association of HR 3.28 (2.00–5.38).	
< 1 year mortality	2497 (2 observational studies)	⊕◯◯◯ Very low^a^,^b^	At 0.9 years, an RR range of 1.00–2.30 and a 95% CI range of 0.60 to 3.80 were reported. At 6 months, an HR of 1.86 (1.29–2.66) was reported.	
2‐year rehospitalization	2099 (1 observational study)	⊕◯◯◯ Very low^b^,^c^	In patients with HFrEF, HFmrEF, and HFpEF, an OR of 0.71 (0.43–1.18), OR of 0.95 (0.58–1.54), and OR of 1.78 (1.09–2.93) were reported, respectively.	
Low BUN 2‐months rehospitalization	1956 (1 observational study)	⊕⊕⊕◯ Moderate^d^	HR 0.74	0.18–3.14
High BUN 2‐months rehospitalization	1956 (1 observational study)	⊕⊕⊕⊕ High	HR 1.23	0.81–1.86
**Cardiac outcomes**				
1‐month Major Adverse Cardiovascular Event	96 (1 observational study)	⊕◯◯◯ Very low^e^,^f^	OR 2.47	1.01–6.01
31‐months Vena cava congestion	103 (1 observational study)	⊕◯◯◯ Very low^e^,^f^	OR 2.98	1.23–7.18
Mean difference NYHA IV ‐ II	108 (1 observational study)	⊕⊕◯◯ Low^d^,^f^	MD 12.03	10.54–13.52
**Renal outcomes**				
1‐year Acute Kidney Injury	371 (1 observational study)	⊕⊕⊕⊕ High	OR 0.90	0.57–1.43
3‐days Acute Kidney Injury	60 (1 observational study)	⊕◯◯◯ Very low^e^,^f^	MD −1.05	−4.78 to 2.68
6.5 years Mean difference between eGFR < 35 and eGFR > 53	355 (1 observational study)	⊕⊕◯◯ Low^e^	MD 2.0	0.22–4.22
1 year Mean difference between eGFR < 60 and eGFR > 60	204 (1 observational study)	⊕◯◯◯ Very low^e^,^f^	MD 1.4	− 3.04 to 5.84

*Note:* GRADE Working Group grades of evidence. **High certainty:** we are very confident that the true effect lies close to that of the estimate of the effect. **Moderate certainty:** we are moderately confident in the effect estimate: the true effect is likely to be close to the estimate of the effect, but there is a possibility that it is substantially different. **Low certainty:** our confidence in the effect estimate is limited: the true effect may be substantially different from the estimate of the effect. **Very low certainty:** we have very little confidence in the effect estimate: the true effect is likely to be substantially different from the estimate of effect. ⊕: One point out of four given. ◯: No point given.

Abbreviations: CI, confidence interval; HR, Hazard Ratio; OR, Odds Ratio; MD, Mean Difference; RR, Risk Ratio.

^a^ Inconsistency due to a general overlap of 95% CI. Therefore, we have downgraded the evidence 1 level.

^b^ Indirectness due to different follow‐up periods in each study. Therefore, we downgraded the evidence 2 levels.

^c^ Indirectness due to different follow‐up periods and populations in each study. Therefore, we downgraded the evidence 2 levels.

^d^ Imprecision due to a wide 95% CI. Therefore, we have downgraded the evidence 1 level.

^e^ Imprecision due to a very wide 95% CI. Therefore, we downgraded the evidence 2 levels.

^f^ Imprecision due to fewer than 300 events. Therefore, we have downgraded the evidence 1 level.

## 4 Discussion

### 4.1 Summary

This review evaluated 20 studies and showed that an elevated BCR is associated with an increased risk of adverse events in heart failure, including mortality, rehospitalization, cardiovascular complications, and renal impairment. On average, an increase in BCR was associated with a higher risk of mortality, especially in long‐term follow‐ups, and with a higher probability of rehospitalization, particularly in patients with preserved ejection fraction. Likewise, patients with high BCR had up to twice the risk of MACE and a tendency toward more cases of hypernatremia and renal dysfunction. Taken together, these findings suggest that elevated BCR is statistically associated with adverse outcomes in heart failure, though its discriminative ability as a prognostic marker is poor. However, the evidence is of low certainty due to variability between studies.

### 4.2 Comparing With SRs or Studies

We found that an increase in BCR was associated with higher mortality and rehospitalization. This utility has already been reported in the review by Paulus et al. [37], who found that an elevated urea/creatinine ratio in critically ill patients was associated with a 29% higher risk of mortality, in addition to indicating muscle protein catabolism and progression to persistent critical illness. In this regard, this same marker has already proven useful by being associated with a threefold increase in mortality risk in patients with HF (9). Although it is not the same marker, it does allow an approximation of the real effect of BCR. Similarly, Tolomeo et al. [38] reported a higher risk of hospitalization (HR 1.13; 95% CI 1.10–1.16) for HF for every five‐unit increase in the BUN/creatinine ratio above 20 in the general population and an even higher risk in patients with HFpEF. Elevated BCR may reflect a state of hemoconcentration, especially when the change in hemoglobin is ≥ 0.8 g/Dl [32], resulting from reduced plasma volume or poor renal perfusion. This, in turn, may explain outcomes such as mortality or rehospitalization, as it indicates an inadequate response to diuretic treatment and increased systemic congestion.

On the other hand, we found that elevated BCR not only predicts mortality but also renal outcomes and cardiovascular events. Similar to the above, this is consistent with previous literature, where an increase in BCR was correlated with worse overall outcomes, including progression of renal dysfunction [39] and an increased risk of venous congestion and long‐term mortality [40]. The increase in the BCR ratio reflects an imbalance between renal perfusion and tubular reabsorption of urea. In scenarios of hypoperfusion or neurohormonal activation (as occurs in advanced heart failure) [41, 42], urea is reabsorbed disproportionately compared to creatinine, which increases the ratio between the two. Therefore, this marker not only reflects renal function but also hemodynamic status and neuroendocrine response, which are factors closely linked to clinical prognosis. The differences between studies could be explained by variations in the type of heart failure (preserved vs. reduced ejection fraction), follow‐up time, average age of the cohorts, and use of optimized treatments. For example, studies with younger patients or those with better therapeutic management tended to show weaker associations, whereas those with more fragile populations or a higher comorbidity burden reported more pronounced effects.

Zhou et al. [43] previously conducted a systematic review and meta‐analysis assessing the prognostic value of the blood urea nitrogen‐to‐creatinine ratio (BCR) in patients with heart failure, encompassing 14 studies published up to October 2023. Their findings indicated that elevated BCR levels were significantly associated with increased all‐cause mortality (HR = 1.67; 95% CI: 1.38–2.00); however, no consistent association was observed with cardiovascular mortality or hospital readmission due to heart failure. Despite these insights, the study presented notable methodological limitations—it did not evaluate the certainty of the evidence, pooled studies with heterogeneous designs and varying follow‐up durations, and included fewer studies than the present review (20 studies). Consequently, our analysis broadens and strengthens the current body of evidence by incorporating a larger dataset, performing a detailed appraisal of methodological quality, and offering an integrated assessment of clinically relevant outcomes. Collectively, these enhancements provide a more comprehensive and reliable understanding of the prognostic role of BCR in heart failure.

### 4.3 Recommendations for Further Research

The main limitations of the available evidence were the heterogeneity in the follow‐up periods, study designs, and patient severity. Therefore, future research should prioritize standardized follow‐ups and control for confounders through the stratification of patients according to heart failure severity, particularly based on ejection fraction phenotypes (HFrEF, HFmrEF, and HFpEF), as well as by comorbidity burden and baseline renal function, which may significantly modify prognostic associations. In addition, an inherently important bias arising from the high frequency of retrospective studies should be addressed by conducting prospective cohorts with longer follow‐ups to better clarify the actual associations. Moreover, further studies in other regions and countries are necessary to generalize the findings, as risk factors, management patterns, and socioeconomic conditions differ across ethnicities and may influence outcomes [44]. Finally, evaluating other associated factors together with BCR should be considered to develop prognostic models for predicting mortality and rehospitalization.

### 4.4 Relevance to Clinical Practice

Although the overall certainty of evidence was low to very low, increased BCR values were consistently linked to higher mortality and rehospitalization, particularly in HFpEF, suggesting potential value as a simple biomarker to identify high‐risk patients and guide closer monitoring and management. In addition, elevated BCR was associated with greater disease severity, including higher NYHA class, increased risk of major adverse cardiovascular events, venous congestion, and impaired renal function. These findings suggest that elevated BCR is statistically associated with higher risk of adverse outcomes; however, with AUC values ranging from 0.57 to 0.61, its ability to discriminate between patients at high and low risk is poor. Finally, BCR provides a low‐cost and accessible tool for risk assessment compared with more expensive biomarkers such as NT‐proBNP, high‐sensitivity troponin, Galectin‐3, or ST2 [45, 46].

### 4.5 Limitations and Strengths

This systematic review presents certain limitations that warrant consideration. A substantial proportion of the included studies were retrospective in nature, which may have introduced residual confounding and reduced the ability to establish causal relationships. In addition, most of the available evidence originated from Asian and high‐income countries, which could limit the external validity and applicability of the findings to broader or underserved populations. Considerable heterogeneity was also observed in the duration of follow‐up across studies, potentially influencing the consistency and comparability of the reported outcomes.

Nevertheless, this review also offers important methodological strengths. It was based on a comprehensive and unrestricted search strategy—without restrictions related to language or publication date—allowing for an inclusive and exhaustive synthesis of the clinical relevance of BCR across diverse heart failure populations. Moreover, the certainty of the evidence was systematically appraised using the GRADE approach, thereby reinforcing the transparency, reliability, and methodological rigor of the analysis.

## 5 Conclusions

The findings of this review indicate that an elevated blood urea to creatinine ratio (BCR) is associated with long‐term mortality in patients with heart failure, particularly among those with preserved ejection fraction. Nonetheless, the overall certainty of the evidence is rated as very low, primarily due to substantial variability across studies and inconsistencies in the follow‐up duration. These limitations constrain the clinical applicability of BCR as a reliable prognostic tool. To strengthen the evidence base, future prospective studies with standardized follow‐up protocols and rigorous adjustment for potential confounding factors are warranted.

## Author Contributions

Miguel Cabanillas‐Lazo, Roger A. Sernaqué‐Mechato, Alvaro Montes‐Baldarrago, Jeancarlo Velazco Muñoz, Valeria Loja Zapata, Ivan Alegre‐Cordero, conceived and designed the study. Roger A. Sernaqué‐Mechato and Carlos Quispe‐Vicuña conducted the literature searches. Roger A. Sernaqué‐Mechato, Alvaro Montes‐Baldarrago, Jeancarlo Velazco Muñoz, Valeria Loja Zapata, and Ivan Alegre‐Cordero screened the search results. Alvaro Montes‐Baldarrago, Jeancarlo Velazco Muñoz, and Valeria Loja Zapata extracted the data. Miguel Cabanillas‐Lazo and Carlos Quispe‐Vicuña performed the data analysis. Miguel Cabanillas‐Lazo, Roger A. Sernaqué‐Mechato, Alvaro Montes‐Baldarrago, Jeancarlo Velazco Muñoz, Valeria Loja Zapata, Ivan Alegre‐Cordero, and Frank Mayta‐Tovalino drafted the manuscript. All authors critically reviewed and edited the final version. All authors had full access to the data and took full responsibility for the decision to submit the manuscript for publication.

## Funding

The authors received no specific funding for this work.

## Ethics Statement

The authors have nothing to report.

## Consent

The authors have nothing to report.

## Conflicts of Interest

The authors declare no conflicts of interest.

## Supporting information

Supporting File

## Data Availability

The data that support the findings of this study are available from the corresponding author upon reasonable request.
