# Supplementary material for: Clinic Value of Blood Urea to Creatinine Ratio in Heart Failure Patients: A Systematic Review
Source: Clin Cardiol. 2026 May 25;49(5):e70344. doi: 10.1002/clc.70344 (PMC13239929; doi:10.1002/clc.70344)
Supplement: Supplementary file 1 — Supporting File [file CLC-49-e70344-s001.docx]

**REVIEW**

**Clinic value of blood urea to creatinine ratio in heart failure patients: A systematic review**

Miguel Cabanillas-Lazo^1^, Roger A Sernaqué-Mechato^2,3^, Alvaro Montes-Baldarrago^4,5^, Jeancarlo Velazco Muñoz^4,5^, Valeria Loja Zapata^4,5^, Ivan Alegre-Cordero^4,5^, Carlos Quispe-Vicuña^1^, Frank Mayta-Tovalino^6^

**Supplementary Material S1. Search strategy**

| Pubmed | #1: Blood Urea Nitrogen[mh] OR Urea*[tiab] OR Bun[tiab] OR ureum[tiab] OR Urea[mh]  #2: Creatinine[mh] OR Creatinin*[tiab] OR Kreatinin*[tiab]  #3: Heart failure[mh] OR “Heart fail*”[tiab] OR “Heart Decompens*”[tiab] OR “heart incompet*”[tiab] OR “heart insuffic*”[tiab] OR “Cardiac Fail*”[tiab] OR “cardiac incompet*”[tiab] OR “cardiac insuffic*”[tiab] OR “cardiac decompens*”[tiab] OR “myocardial insuffic*”[tiab] OR “Myocardial Fail*”[tiab] OR “myocardial Decompens*”[tiab] OR “myocardial incompet*”[tiab] OR “cardial fail*”[tiab] OR  “cardial decompens*”[tiab] OR “cardial insuffic*”[tiab] OR “cardial incompet*”[tiab] OR “decompensatio cordis” [tiab] OR “insufficientia cardis*”[tiab]  #4: #1 and #2 and #3 |
| --- | --- |
| Scopus | #1: TITLE-ABS(Urea* OR Bun OR ureum) OR AUTHKEY(Urea* OR Bun OR ureum)  #2: TITLE-ABS(Creatinin* OR Kreatinin*) OR AUTHKEY(Creatinin* OR Kreatinin*)  #3: TITLE-ABS( “Heart fail*” OR “Heart Decompens*” OR “heart incompet*” OR “heart insuffic*” OR “Cardiac Fail*” OR “cardiac incompet*” OR “cardiac insuffic*” OR “cardiac decompens*” OR “myocardial insuffic*” OR “Myocardial Fail*” OR “myocardial Decompens*” OR “myocardial incompet*” OR “cardial fail*” OR  “cardial decompens*” OR “cardial insuffic*” OR “cardial incompet*” OR “decompensatio cordis” OR “insufficientia cardis*”) OR AUTHKEY( “Heart fail*” OR “Heart Decompens*” OR “heart incompet*” OR “heart insuffic*” OR “Cardiac Fail*” OR “cardiac incompet*” OR “cardiac insuffic*” OR “cardiac decompens*” OR “myocardial insuffic*” OR “Myocardial Fail*” OR “myocardial Decompens*” OR “myocardial incompet*” OR “cardial fail*” OR “cardial decompens*” OR “cardial insuffic*” OR “cardial incompet*” OR “decompensatio cordis” OR “insufficientia cardis*”)  #4: #1 and #2 and #3 |
| Web of Science | #1: TI=(Urea* OR Bun OR ureum) OR AB=(Urea* OR Bun OR ureum) OR AK=(Urea* OR Bun OR ureum) OR KP=(Urea* OR Bun OR ureum) OR TS=(Urea* OR Bun OR ureum)  #2: TI=(Creatinin* OR Kreatinin*) OR AB=(Creatinin* OR Kreatinin*) OR AK=(Creatinin* OR Kreatinin*) OR KP=(Creatinin* OR Kreatinin*) OR TS=(Creatinin* OR Kreatinin*)  #3: TI=( “Heart fail*” OR “Heart Decompens*” OR “heart incompet*” OR “heart insuffic*” OR “Cardiac Fail*” OR “cardiac incompet*” OR “cardiac insuffic*” OR “cardiac decompens*” OR “myocardial insuffic*” OR “Myocardial Fail*” OR “myocardial Decompens*” OR “myocardial incompet*” OR “cardial fail*” OR  “cardial decompens*” OR “cardial insuffic*” OR “cardial incompet*” OR “decompensatio cordis” OR “insufficientia cardis*”) OR AB=( “Heart fail*” OR “Heart Decompens*” OR “heart incompet*” OR “heart insuffic*” OR “Cardiac Fail*” OR “cardiac incompet*” OR “cardiac insuffic*” OR “cardiac decompens*” OR “myocardial insuffic*” OR “Myocardial Fail*” OR “myocardial Decompens*” OR “myocardial incompet*” OR “cardial fail*” OR “cardial decompens*” OR “cardial insuffic*” OR “cardial incompet*” OR “decompensatio cordis” OR “insufficientia cardis*”) OR AK=( “Heart fail*” OR “Heart Decompens*” OR “heart incompet*” OR “heart insuffic*” OR “Cardiac Fail*” OR “cardiac incompet*” OR “cardiac insuffic*” OR “cardiac decompens*” OR “myocardial insuffic*” OR “Myocardial Fail*” OR “myocardial Decompens*” OR “myocardial incompet*” OR “cardial fail*” OR “cardial decompens*” OR “cardial insuffic*” OR “cardial incompet*” OR “decompensatio cordis” OR “insufficientia cardis*”) OR KP=( “Heart fail*” OR “Heart Decompens*” OR “heart incompet*” OR “heart insuffic*” OR “Cardiac Fail*” OR “cardiac incompet*” OR “cardiac insuffic*” OR “cardiac decompens*” OR “myocardial insuffic*” OR “Myocardial Fail*” OR “myocardial Decompens*” OR “myocardial incompet*” OR “cardial fail*” OR “cardial decompens*” OR “cardial insuffic*” OR “cardial incompet*” OR “decompensatio cordis” OR “insufficientia cardis*”) OR TS=( “Heart fail*” OR “Heart Decompens*” OR “heart incompet*” OR “heart insuffic*” OR “Cardiac Fail*” OR “cardiac incompet*” OR “cardiac insuffic*” OR “cardiac decompens*” OR “myocardial insuffic*” OR “Myocardial Fail*” OR “myocardial Decompens*” OR “myocardial incompet*” OR “cardial fail*” OR “cardial decompens*” OR “cardial insuffic*” OR “cardial incompet*” OR “decompensatio cordis” OR “insufficientia cardis*”)  #4: #1 and #2 and #3 |
| Google Scholar | (bun to creatinine) AND ("heart failure") |
| Embase | ('urea'/mj OR 'alphadrate':ti,ab,kw OR 'aquacare':ti,ab,kw OR 'aquadrate':ti,ab,kw OR 'aqurea':ti,ab,kw OR 'balisa':ti,ab,kw OR 'banjil':ti,ab,kw OR 'basodexan':ti,ab,kw OR 'calmurid':ti,ab,kw OR 'calmuril':ti,ab,kw OR 'carbamide':ti,ab,kw OR 'carbamide cream':ti,ab,kw OR 'carbonamide':ti,ab,kw OR 'carbonyldiamide':ti,ab,kw OR 'carmed':ti,ab,kw OR 'carmol':ti,ab,kw OR 'carmol-40':ti,ab,kw OR 'elacutan':ti,ab,kw OR 'elaqua xx':ti,ab,kw OR 'epimide 50':ti,ab,kw OR 'euderm':ti,ab,kw OR 'ferzea':ti,ab,kw OR 'gordons urea':ti,ab,kw OR 'hyanit':ti,ab,kw OR 'keratinamin':ti,ab,kw OR 'laceran':ti,ab,kw OR 'linola':ti,ab,kw OR 'nubral creme':ti,ab,kw OR 'nutraplus':ti,ab,kw OR 'onychomal':ti,ab,kw OR 'soft u derm':ti,ab,kw OR 'sterile urea':ti,ab,kw OR 'ultramide':ti,ab,kw OR 'urea':ti,ab,kw OR 'urea cream':ti,ab,kw OR 'urea rea':ti,ab,kw OR 'urea solution':ti,ab,kw OR 'ureaphil':ti,ab,kw OR 'ureate':ti,ab,kw OR 'urecare':ti,ab,kw OR 'uremol':ti,ab,kw OR 'ureophil':ti,ab,kw OR 'urepearl':ti,ab,kw OR 'ureum':ti,ab,kw OR 'urevert':ti,ab,kw OR 'uricrim':ti,ab,kw OR 'vanamide':ti,ab,kw OR 'urea blood level'/mj OR 'blood urea':ti,ab,kw OR 'plasma urea':ti,ab,kw OR 'serum urea':ti,ab,kw OR 'serum ureum':ti,ab,kw OR 'urea blood level':ti,ab,kw OR 'urea serum level':ti,ab,kw OR 'urea, blood':ti,ab,kw OR 'urea, plasma':ti,ab,kw OR 'urea nitrogen blood level'/mj OR 'bun':ti,ab,kw OR 'blood urea nitrogen':ti,ab,kw OR 'plasma urea nitrogen':ti,ab,kw OR 'serum bun':ti,ab,kw OR 'serum urea nitrogen':ti,ab,kw OR 'urea nitrogen blood level':ti,ab,kw) AND ('creatinine'/mj OR '1 methylglycocyamidine':ti,ab,kw OR '1 methylhydantoin 1 imide':ti,ab,kw OR '2 imino 1 methyl 4 imidazolinone':ti,ab,kw OR 'creatinin':ti,ab,kw OR 'creatinine':ti,ab,kw OR 'creatinine hydrochloride':ti,ab,kw OR 'kreatinine':ti,ab,kw OR 'methylglycocyamimine':ti,ab,kw OR 'creatinine blood level'/mj OR 'blood creatinine':ti,ab,kw OR 'creatinine blood level':ti,ab,kw OR 'creatinine, serum':ti,ab,kw OR 'plasma creatinine':ti,ab,kw OR 'serum creatinine':ti,ab,kw) AND ('heart failure'/mj OR 'backward failure, heart':ti,ab,kw OR 'cardiac backward failure':ti,ab,kw OR 'cardiac decompensation':ti,ab,kw OR 'cardiac failure':ti,ab,kw OR 'cardiac incompetence':ti,ab,kw OR 'cardiac insufficiency':ti,ab,kw OR 'cardiac stand still':ti,ab,kw OR 'cardial decompensation':ti,ab,kw OR 'cardial insufficiency':ti,ab,kw OR 'chronic heart failure':ti,ab,kw OR 'chronic heart insufficiency':ti,ab,kw OR 'decompensatio cordis':ti,ab,kw OR 'decompensation, heart':ti,ab,kw OR 'heart backward failure':ti,ab,kw OR 'heart decompensation':ti,ab,kw OR 'heart failure':ti,ab,kw OR 'heart incompetence':ti,ab,kw OR 'heart insufficiency':ti,ab,kw OR 'insufficientia cardis':ti,ab,kw OR 'myocardial failure':ti,ab,kw OR 'myocardial insufficiency':ti,ab,kw) |

**Supplementary Material S2. Articles excluded**

| **Study** | **Author, year** | **Reason for exclusion** |
| --- | --- | --- |
| Influence of renal dysfunction phenotype on mortality in decompensated heart failure with preserved and mid-range ejection fraction | Casado, 2017 | OTHER EXPOSITION |
| Overestimation of glomerular filtration rate by creatinine-based equation in heart failure patients is predicted by a novel scoring system | Ishigo, 2020 | OTHER OUTCOME |
| Persistent high blood urea nitrogen level is associated with increased risk of cardiovascular events in patients with acute heart failure | Jujo, 2017 | OTHER EXPOSITION |
| Prognostic significance of creatinine increases during an acute heart failure admission in patients with and without residual congestion: a post hoc analysis of the PROTECT data | Metra, 2018 | OTHER EXPOSITION |
| Exercise gas exchange in continuous-flow left ventricular assist device recipients | Mezzani, 2018 | OTHER EXPOSITION |
| Prognostic Impact of Blood Urea Nitrogen Changes During Hospitalization in Patients With Acute Heart Failure Syndrome | Miura, 2013 | OTHER OUTCOME |
| The Effect of Furosemide Dose Administered in the Out-of-hospital Setting on Renal Function Among Patients with Suspected Acute Decompensated Heart Failure | Nieves, 2015 | OTHER EXPOSITION |
| Prognostic Value of Fractional Excretion of Urea Nitrogen at Discharge in Acute Decompensated Heart Failure | Nogi, 2021 | OTHER EXPOSITION |
| Basic laboratory parameters as predictors of in-hospital death in patients with acute decompensated heart failure: data from a large single-center cohort | Ostrowska, 2017 | OTHER EXPOSITION |
| Heartbeat:Blood urea nitrogen to creatinine ratio predicts outcome in acute heart failure | Otto, 2017 | OTHER TYPE OF STUDY |
| Predictive value of blood urea nitrogen/creatinine ratio in the long-term prognosis of patients with acute myocardial infarction complicated with acute heart failure | Qian, 2019 | OTHER POPULATION |
| Plasma Levels of Intact Parathyroid Hormone and Congestion Burden in Heart Failure: Clinical Correlations and Prognostic Role | Scicchitano, 2022 | OTHER EXPOSITION |
| The Urea-to-Creatinine Ratio Is Predictive of Worsening Kidney Function in Ambulatory Heart Failure Patients | Sood, 2015 | OTHER EXPOSITION |
| Interaction Between Loop Diuretic-Associated Mortality and Blood Urea Nitrogen Concentration in Chronic Heart Failure | Testani, 2011 | OTHER EXPOSITION |
| A combined-biomarker approach to clinical phenotyping renal dysfunction in heart failure | Testani, 2014 | OTHER EXPOSITION |
| Diagnostic Values of Blood Urea Nitrogen (BUN), Creatinine (Cr), and the Ratio of BUN to Cr for Distinguishing Heart Failure from Asthma and Chronic Obstructive Pulmonary Disease | Zhang, 2022 | OTHER POPULATION |
| Serum biochemical determinants of peripheral congestion assessed by bioimpedance vector analysis in acute heart failureSerum biochemical determinants of peripheral congestion assessed by bioimpedance vector analysis in acute heart failure | Massari, 2019 | OTHER OUTCOME |

**Supplementary Material S3**. Characteristics of included studies (n=20)

|  | **Country** | **Study  design** | **Main Inclusion criteria** | **Main exclusion criteria** | **Sample size** | **Subgroups in Heart Failure** | **Age (years)** | **Sex (male %)** | **Control criteria** | **Outcome measure** | **Cutt-off value** | **Blood sample time** | **Follow up time** | **Funder** |
| --- | --- | --- | --- | --- | --- | --- | --- | --- | --- | --- | --- | --- | --- | --- |
|  | USA | Cohort study | HF patients | Patients not discharged alive | 716 | No hyponatremia Persistent Hyponatremia Descompensation hyponatremia  Treatment-induced hyponatremia | No hyponatremia:  62 ± 14 Persistent Hyponatremia:  56 ± 14 Descompensation hyponatremia:  58 ± 15 Treatment-induced hyponatremia:  56 ± 15 | No hyponatremia:  71% Persistent Hyponatremia:  81% Descompensation hyponatremia:  91% Treatment-induced hyponatremia:  72% | NR | Prognosis | NR | NR | NR | Research Foundation – Flanders Foundation Limburg Sterk Merk and the National Institutes of Health grant |
|  | Israel | Cohort study | HF patients | Hemodialysis | 355 | Tertile 1 (eGFR < 35) Tertile 2 (eGFR 35-53) Tertile 3 (eGFR 953) | Tertile 1 (eGFR < 35) 75 ± 10 Tertile 2 (eGFR 35-53) 77 ± 11 Tertile 3 (eGFR 953) 70 ± 14 | Tertile 1 (eGFR < 35) 55 (47%) Tertile 2 (eGFR 35-53) 65 (55%) Tertile 3 (eGFR 953) 68 (58%) | NR | Prognosis | 25 | Measured at baseline | 6.5 years | None |
|  | Egypt | Case control study | ADHF patients | Pregnancy, kidney disease or obstructive uropathy | 60 | Group I:  non-AKI with low BCR Group II:  non-AKI with high BCR Group III:  AKI with low BCR  Group IV:  AKI with high BCR | 62.8 ± 10.4 | 43 (71.7%) | NR | Prognosis | 17.4 | Measured at baseline, on day two and before discharge | NR | None |
|  | Italy | Cohort study | HF patients and RD | Patients hospitalized within more 3 months for HF, other cardiac, renal, blood or autoimmune diseases | 103 | Collapse Index > 50 % Collapse Index < 50 % Inferior vena cava < 2 cm Inferior vena cava > 2 cm No Caval Congestion Caval Congestion | Collapse Index > 50 %: 73.8 ± 7.7 Collapse Index < 50 % 75.2 ± 8.2 Inferior vena cava < 2 cm 73.5 ± 7.8 Inferior vena cava > 2 cm 75.8 ± 8 No Caval Congestion 74.1 ± 8.0 Caval Congestion 75.2 ± 7.9 | Collapse Index > 50 %: 24 (42.8%) Collapse Index < 50 % 21 (44.6%) Inferior vena cava < 2 cm 27 (43.5%) Inferior vena cava > 2 cm 18 (43.9%) No Caval Congestion 28 (42.4%) Caval Congestion 17 (45.9%) | NR | Prognosis | 25.5 | Measured at baseline | 31 months | None |
|  | Indonesia | Cohort study | AHF patients | Acute coronary syndrome, congenital heart disease, malignancy or chronic kidney disease | 96 | Patients with MACEs Patients with no MACEs | Patients with MACEs: 59.0 ± 9.68 Patients with no MACEs: 57.4 ± 7.97 | Patients with MACEs: 36 (47.4 %) Patients with no MACEs: 40 (52.6 %) | NR | Prognosis | 16.05 | Measured at baseline | 1 month | None |
|  | Netherlands | Cohort study | AHF patients | Non PROTECT trial patients | 2033 | BUN/creatinine ratio higher than normal range BUN/creatinine ratio within normal range BUN/creatinine ratio lower than normal range | BUN/creatinine ratio higher than normal range 69±12 BUN/creatinine ratio within normal range 71±11 BUN/creatinine ratio lower than normal range 70±12 | BUN/creatinine ratio higher than normal range 342 (71%) BUN/creatinine ratio within normal range 948 (66%) BUN/creatinine ratio lower than normal range 17 (61%) | NR | Prognosis | 17.5 | Measured at baseline | NR | None |
|  | Japan | Cohort study | ADHF patients | Acute coronary syndrome | 2090 | Extreme haemodilution Modest haemodilution Modest haemoconcentration Extreme haemoconcentration | Extreme haemodilution: 75.0 (63.0 - 83.0) Modest haemodilution: 76.0 (67.0 - 84.0) Modest haemoconcentration: 77.0 (67.0 - 83.0) Extreme haemoconcentration: 75.0 (63.0 - 82.0) | Extreme haemodilution: 309 (64.5%) Modest haemodilution: 337 (60.7%) Modest haemoconcentration: 308 (60.8%) Extreme haemoconcentration: 340 (61.8%) | NR | Prognosis | 22.1 | Measured at baseline | 771 days | Grant-in-Aid for Young Scien- tists, Grant-in-Aid for Scientific Research, Health Labour Sciences Research Grant, |
|  | China | Cohort study | CHF patients | Svere cardiac function impairment, hemodyalisis or systemic diseases | 504 | BCR < 19.37 BCR ≥ 19.37 | BCR < 19.37: 77 (71, 81) BCR ≥ 19.37 76 (68, 81) | BCR < 19.37: 151 (53.9%) BCR ≥ 19.37 109 (48.7%) | NR | Prognosis | 19.37 | Measured at baseline | 1 year | Beijing Natural Science Foundation |
|  | USA | Case control study | HF patients | Recent myocardial infaction, cardiogenic shock or cardiomiopathies | 540 | Comparative PRECEDENT study | Comparative: 64 ± 13 PRECEDENT study: 61 ± 14 | Comparative: 207 (68%) PRECEDENT study: 170 (72%) | Symptomatic heart failure with one or more vasoactive drugs | Prognosis | 27 | Measured at baseline | 343 ± 185 days | None |
|  | Italy | Cohort study | HF patients | Acute coronary syndrome, cardiac surgery, malignancy | 436 | NR | 75±11 | 227 (52%) | NR | Prognosis | 25 | Measured at baseline | 536 days | None |
|  | Japan | Prospective multicenter study | HF patients | Patients with anuria, disturbed consciousness or pregnancy | 3349 | Hypernatremic event No hypernatremic event | Hypernatremic event: 80.1 ± 11.5 No hypernatremic event: 77.2 ± 12.4 | Hypernatremic event: 58 (55.2 %) No hypernatremic event: 1567 (56.6%) | NR | Prognosis | 17.8 | Measured at baseline | NR | Otsuka Pharmacutical |
|  | España | Cohort study | HF patients | Admitted to intensive care unit, valvular heart disease | 203 | Survivors Deceased | Survivors: 78.0 ± 8.6 Deceased: 82.1 ± 5.8 | Survivors: 72 (47.4 %) Deceased: 20 (47.6%) | NR | Prognosis | NR | Measured at baseline | 1 year | None |
|  | USA | Case control study | HF patients | Any disease suspected to shorten survival | 6439 | No proteinuria Proteinuria | No proteinuria: 59.3 ± 10.1 Proteinuria: 59.7 ± 10.4 | No proteinuria: 4113 (86.1%) Proteinuria: 1411 (84.9%) | Heart failure diagnosis and medical treatment | Prognosis | 17.3 | Measured at baseline | 2.8 years | National Institutes of Health |
|  | USA | Retrospective cohort | HF patients | Patients on renal replacement therapy or those admitted to intervntional cardiology service | 896 | High BCR>20  Low BCR<20 | High BCR:  68.5 ± 13.8  Low BCR:  59.6 ± 16.0 | High BCR: 325 (50.8%)  Low BCR: 571 (56.6%) | NR | Prognosis | 20.0 | Measured at baseline | 2.6 years | National Institutes of Health |
|  | España | Cohort study | HF patients | Life expectancy less than six months, malignancy or CKD | 204 | FGe < 60mL/min/1,73m2:  FGe ≥ 60 mL/min/1,73m2:  BCR ratio ≤ 50  BCR ratio > 50 | FGe < 60mL/min/1,73m2: 82 (7) FGe ≥ 60 mL/min/1,73m2: 79 (8) BCR ratio ≤ 50 80 (10) BCR ratio > 50 82 (8) | FGe < 60mL/min/1,73m2: 63 (52,5%) FGe ≥ 60 mL/min/1,73m2: 40 (47,6%) BCR ratio ≤ 50 NR BCR ratio > 50 NR | NR | Prognosis | 22 | Measured at baseline | 1 year | Instituto de Salud Carlos III, Ministerio de Economía y Competitividad |
|  | Japan | Cohort study | HF patients | Cardiac surgery, hemodyalisis, malignancy | 751 | High BCR  Low BCR | High BCR: 72.4 ± 12.8 Low BCR: 67.9 ± 17.8 | High BCR: 154 (55.2%) Low BCR: 207 (74.5%) | NR | Prognosis | 20.4 | Measured at baseline | 1.9 years | Grant-in-Aid for Scientifc Research |
|  | China | Cohort study | HF patients | Tuberculosis, malignancy, diagnosed old guidelines | 2099 | HFrEH with Low BCR: HFrEH HighBCR: HFmrEF Low BCR: HFmrEF HighBCR: HPpEF Low BCR: HPpEF HighBCR: | HFrEH with Low BCR: 66 (58–76) HFrEH HighBCR: 70 (61–79) HFmrEF Low BCR: 72 (62–80)HFmrEF HighBCR: 74 (64–81) HPpEF Low BCR: 74 (64–81) HPpEF HighBCR: 72 (63–82) | HFrEH with Low BCR: 496 (62.4%) HFrEH HighBCR: 63 (41.7%) HFmrEF Low BCR: 381 (63.4%) HFmrEF HighBCR: 71 (55.5%) HPpEF Low BCR: 228 (67.7%) HPpEF HighBCR: 63 (56.8%) | NR | Prognosis | 20.4 | Measured at baseline | 2 years | Foundation of medical research major Project of Tianjin Heart Association |
|  | Japan | Cohort study | HF patients | Cardiac surgery, invasive procedures, on hemodyalisis | 371 | Non-AKI+low BCR: Non-AKI+high BCR: AKI+low BCR: AKI+high BCR: | Non-AKI+low BCR: 71±13 Non-AKI+high BCR: 75±12 AKI+low BCR: 73±11 AKI+high BCR: 76±12 | Non-AKI+low BCR: 104 (78%) Non-AKI+high BCR: 56 (40%) AKI+low BCR: 32 (63%) AKI+high BCR: 29 (60%) | NR | Prognosis | 22.1 | Measured at baseline | 12 months | Program for Promotion of Fundamental Studies in Health Sciences of the Pharmaceuticals and Medical Devices Agency |
|  | USA | Cohort study | HF patients | NR | 44 | NR | NR | NR | NR | Severity | NR | Measured at baseline | NR | CRC Grant RR-47, from the National Institutes of Health |
|  | China | Case control study | HF patients | Severe liver or kidney disfunction, anemia, acute myocardial infarction, pregnancy | 108 | Stage II Stage III Stage IV Control | Stage II: 52.87 ± 9.98 Stage III: 53.67 ± 9.92 Stage IV: 54.58 ± 9.83 Control: 53.06 ± 9.39 | Stage II: 14 (60.87%) Stage III: 39 (60 %) Stage IV: 13 (65 %) Control: 62 (53.91 %) | Normal electriocardiogram and no history of cardiovascular disease | Severity | NR | Measured at baseline | NR | None |

**Supplementary Material S4**. The Newcastle-Ottawa Scale (NOS) for Assessing the Quality of Nonrandomized Studies in Meta-Analysis

| **COHORT STUDY** | **SELECTION** | | | | **COMPARABILITY** | **EXPOSURE** | | |  |
| --- | --- | --- | --- | --- | --- | --- | --- | --- | --- |
| **Item** | **Representativeness of the exposed cohort** | **Selection of the non exposed cohort** | **Ascertainment of exposure** | **Definition of cohorts** | **Comparability of cohorts on the basis of design or analysis** | **Assessment of outcome** | **Was follow-up long enough for outcomes to occur** | **Adequacy of follow up of cohorts** | **Risk of bias assessment** |
| Verbrugge, 2016 |  |  | * |  | * | * | * | * | Moderate risk |
| Gotsman, 2010 |  | * | * | * | * | * | * |  | Low risk |
| Parrinello, 2015 | * | * | * | * | * | * | * |  | Low risk |
| Alya, 2019 | * | * | * | * | * | * | * |  | Low risk |
| Matsue, 2017 | * | * | * | * | * | * | * |  | Low risk |
| Sujino, 2019 | * | * | * | * | * | * | * | * | Low risk |
| Brisco, 2016 | * | * | * | * | * | * | * | * | Low risk |
| Brisco, 2013 | * | * | * | * | * | * | * |  | Low risk |
| Wang, 2023 | * | * | * | * | * | * | * | * | Low risk |
| Aronson, 2004 | * | * | * |  | * | * | * | * | Low risk |
| Massari, 2020 | * | * | * |  | * | * | * | * | Low risk |
| Kinugawa, 2017 |  |  | * | * | * | * |  |  | Moderate risk |
| Rubio-garcía, 2019 | * | * | * | * | * | * |  | * | Low risk |
| Josa-laorden, 2018 | * |  | * | * | * | * | * | * | Low risk |
| Murata, 2018 | * | * | * |  | * | * | * |  | Low risk |
| Kang, 2022 | * |  | * | * | * | * | * |  | Low risk |
| Takaya, 2016 | * | * | * | * | * | * | * |  | Low risk |
| Sakr, 2023 | * |  | * |  | ** | * |  | * | Low risk |
| Schaer, 1983 | * |  | * |  | * |  |  |  | High risk |
| Xie, 2023 | * | * | * | * | ** | * |  |  | Low risk |
